# 2-Amino-4-phenyl-5,6-dihydro­benzo[*h*]quinoline-3-carbonitrile–3-amino-1-phenyl-9,10-dihydro­phenanthrene-2,4-dicarbonitrile (5/3)

**DOI:** 10.1107/S1600536811040505

**Published:** 2011-10-08

**Authors:** Abdullah M. Asiri, Abdulrahman O. Al-Youbi, Hassan M. Faidallah, Seik Weng Ng

**Affiliations:** a Chemistry Department, Faculty of Science, King Abdulaziz University, PO Box 80203 Jeddah, Saudi Arabia; b Center of Excellence for Advanced Materials Research, King Abdulaziz University, PO Box 80203 Jeddah, Saudi Arabia; c Department of Chemistry, University of Malaya, 50603 Kuala Lumpur, Malaysia

## Abstract

The asymmetric unit of the 5:3 title co-crystal of 2-amino-4-phenyl-5,6-dihydro­benzo[*h*]quinoline-3-carbonitrile and 3-amino-1-phenyl-9,10-dihydro­phenanthrene-2,4-dicarbonitrile, 0.625C_20_H_15_N_3_.0.375C_22_H_15_N_3_, has the atoms of the fused-ring system and those of the amino, cyano and phenyl substitutents overlapped. The fused-ring system is buckled owing to the ethyl­ene linkage in the central ring, the two flanking aromatic rings being twisted by 20.1 (1)°. This ethyl­ene portion is disordered over two positions in a 1:1 ratio. The phenyl ring is twisted by 69.5 (1)° relative to the amino- and cyano-bearing aromatic ring. In the crystal, two mol­ecules are linked by an N—H⋯N hydrogen bond, generating a a helical chain along [010].

## Related literature

For the synthesis, see: Aly *et al.* (1991 ▶); Paul *et al.* (1998 ▶). For related structures, see: Asiri *et al.* (2011*a* ▶,*b* ▶).
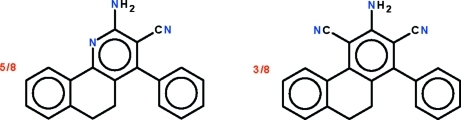


## Experimental

### 

#### Crystal data

0.625C_20_H_15_N_3_·0.375C_22_H_15_N_3_
*M* *_r_* = 306.36
Orthorhombic, 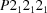

*a* = 6.9611 (2) Å
*b* = 12.6093 (2) Å
*c* = 17.4933 (3) Å
*V* = 1535.47 (6) Å^3^
*Z* = 4
Cu *K*α radiation
μ = 0.62 mm^−1^
*T* = 100 K
0.30 × 0.20 × 0.02 mm

#### Data collection

Agilent SuperNova Dual diffractometer with an Atlas detector
Absorption correction: multi-scan (*CrysAlis PRO*; Agilent, 2010 ▶) *T*_min_ = 0.835, *T*_max_ = 0.988
6293 measured reflections
1794 independent reflections
1707 reflections with *I* > 2σ(*I*)
*R*_int_ = 0.018

#### Refinement

*R*[*F* ^2^ > 2σ(*F* ^2^)] = 0.040
*wR*(*F* ^2^) = 0.119
*S* = 1.05
1794 reflections
240 parameters
24 restraints
H atoms treated by a mixture of independent and constrained refinement
Δρ_max_ = 0.19 e Å^−3^
Δρ_min_ = −0.23 e Å^−3^

### 

Data collection: *CrysAlis PRO* (Agilent, 2010 ▶); cell refinement: *CrysAlis PRO*; data reduction: *CrysAlis PRO*; program(s) used to solve structure: *SHELXS97* (Sheldrick, 2008 ▶); program(s) used to refine structure: *SHELXL97* (Sheldrick, 2008 ▶); molecular graphics: *X-SEED* (Barbour, 2001 ▶); software used to prepare material for publication: *publCIF* (Westrip, 2010 ▶).

## Supplementary Material

Crystal structure: contains datablock(s) global, I. DOI: 10.1107/S1600536811040505/zs2145sup1.cif

Structure factors: contains datablock(s) I. DOI: 10.1107/S1600536811040505/zs2145Isup2.hkl

Supplementary material file. DOI: 10.1107/S1600536811040505/zs2145Isup3.cml

Additional supplementary materials:  crystallographic information; 3D view; checkCIF report

## Figures and Tables

**Table 1:** Hydrogen-bond geometry (Å, °)

*D*—H⋯*A*	*D*—H	H⋯*A*	*D*⋯*A*	*D*—H⋯*A*
N2—H1⋯N3^i^	0.88 (1)	2.37 (2)	3.175 (2)	152 (3)

Symmetry code: (i) 
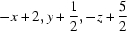
.

**Fig. 1.**
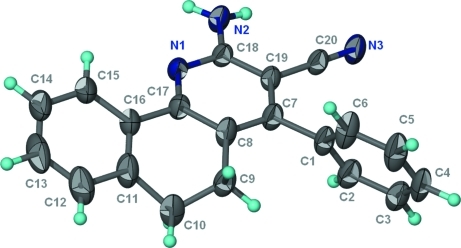
Thermal ellipsoid plot (Barbour, 2001) of C20H15N3 at the 70% probability level. Hydrogen atoms are drawn as spheres of arbitrary radius.

**Fig. 2.**
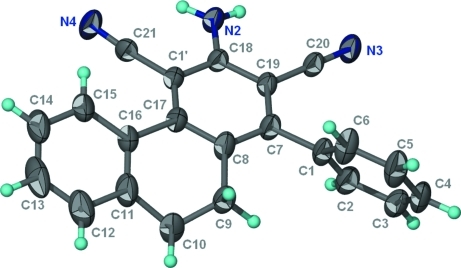
Thermal ellipsoid plot (Barbour, 2001) of C22H15N3 at the 70% probability level. Hydrogen atoms are drawn as spheres of arbitrary radius.

**Fig. 3.**
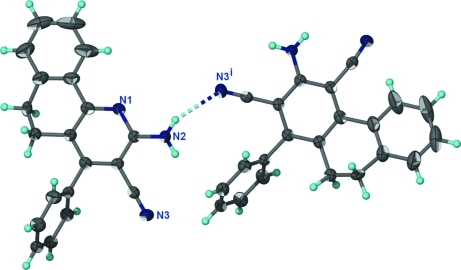
Thermal ellipsoid plot (Barbour, 2001) of C20H15N3 (62.5% component) and C22H15N3 (37.5% component) related by twofold screw axial symmetry. For symmetry code (i), see Table 1.

## supplementary crystallographic information

### Comment

2-Amino-4-phenyl-5,6-dihydrobenzoquinoline-3-carbonitrile is synthesized from the reaction of the α-substituted cinnamonitrile, C_6_H_5_CH═C(CN)_2_, with α-tetralone in a reaction that is catalyzed by ammonium acetate (Aly *et al.*, 1991). The synthesis when conducted under microwave irradiation leads to an improved yield (Paul *et al.*, 1998). In previous studies, we obtained instead di-carbonitrile substituted dihydrophenanthrenes (3-amino-1-(4-methoxyphenyl)-9,10- dihydrophenanthrene-2,4-dicarbonitrile and 3-amino-1-(2*H*-1,3-benzodioxol-5-yl)- 9,10-dihydrophenanthrene-2,4-dicarbonitrile) with 4-methoxybenzaldehyde and piperonaldehyde in syntheses that differed slightly from the reported ones as we used substituted benzaldehydes, α-tetralone and ethyl cyanoacetate along with a molar excess of ammonium acetate (Asiri *et al.*, 2011*a*; 2011*b*).

In this study, the reaction of benzaldehyde, α-tetralone and ethyl cyanoacetate yielded the co-crystal of 2-amino-4-phenyl-5,6-dihydrobenzoquinoline-3-carbonitrile (C_20_H_15_N_3_) and 3-amino-1-phenyl-9,10-dihydrophenanthrene-2,4-dicarbonitrile (C_22_H_15_N_3_), with the two components present in a 5: 3 molar ratio (Scheme I). The fused-ring system is buckled owing to the ethylene linkage in the central ring with the two flanking aromatic rings twisted by 20.1 (1)°. Relative to the amino- and cyano-bearing aromatic ring, the phenyl ring is twisted by 69.5 (1) ° (Fig. 1 and Fig. 2). Two molecules are linked by an N—H···N hydrogen bond to generate a helical chain (Table 1 and Fig. 3). The ethylene portion is disordered over two positions in a 1:1 ratio.

### Experimental

A mixture of benzaldehyde (1.06 g,10 mmol), α-tetralone (1.46 g, 10 mmol), ethyl cyanoacetate (1.13 g, 10 mmol) and ammonium acetate (6.16 g, 80 mmol) in absolute ethanol (50 ml) was refluxed for 6 h. The mixture was allowed to cool and the precipitate that formed was filtered, washed with water, dried and recrystallized from DMF.

### Refinement

Carbon-bound H-atoms were placed in calculated positions [C–H = 0.95–0.99 Å; *U*_iso_(H) 1.2*U*_eq_(C)] and were included in the refinement in the riding model approximation. The amino H-atoms were located in a difference Fourier map and were refined with a distance restraint of N—H = 0.88±0.01 Å and with their isotropic displacement parameters refined.

The crystal is a co-crystal of 2-amino-4-phenyl-5,6-dihydrobenzoquinoline-3-carbonitrile (C_20_H_15_N_3_) and 3-amino-1-phenyl-9,10-dihydrophenanthrene-2,4-dicarbonitrile (C_22_H_15_N_3_). The first component is a dihydrobenzoquinoline and has only one cyano substituent. The second component is a dihydrophenanthrene with two cyano substituents. The two-coordinate N atom of the first molecule occupies the same site as the three-coordinate C atom of the second molecule. As the occupancy refined to an almost 5:3 ratio, the occupancy was then fixed as this ratio. The ethylene –CH_2_CH_2_– portion (whose atoms lie on general positions) is disordered over two sites. The occupancy could not be refined, and was fixed as 1:1. The 1,2-connected carbon-carbon distances were restrained to 1.54±0.01 Å and the 1,3-related ones to 2.51±0.01 Å. The displacement parameters of the primed atoms were set to those of the unprimed ones, and the were restrained to be nearly isotropic. Despite the use of low temperature, copper radiation, long exposure times and a large number of redundant reflections, the Flack parameter could not be refined. 1252 Friedel pairs were merged.

### Crystal data

**Table:**

0.625C_20_H_15_N_3_·0.375C_22_H_15_N_3_	*F*(000) = 642
*M**_r_* = 306.36	*D*_x_ = 1.325 Mg m^−^^3^
Orthorhombic, *P*2_1_2_1_2_1_	Cu *K*α radiation, λ = 1.54184 Å
Hall symbol: P 2ac 2ab	Cell parameters from 3717 reflections
*a* = 6.9611 (2) Å	θ = 3.5–74.3°
*b* = 12.6093 (2) Å	µ = 0.62 mm^−^^1^
*c* = 17.4933 (3) Å	*T* = 100 K
*V* = 1535.47 (6) Å^3^	Plate, brown-orange
*Z* = 4	0.30 × 0.20 × 0.02 mm

### Data collection

**Table:**

Agilent SuperNova Dual diffractometer with an Atlas detector	1794 independent reflections
Radiation source: SuperNova (Cu) X-ray Source	1707 reflections with *I* > 2σ(*I*)
Mirror	*R*_int_ = 0.018
Detector resolution: 10.4041 pixels mm^-1^	θ_max_ = 74.5°, θ_min_ = 4.3°
ω scans	*h* = −8→7
Absorption correction: multi-scan (*CrysAlis PRO*; Agilent, 2010)	*k* = −13→15
*T*_min_ = 0.835, *T*_max_ = 0.988	*l* = −21→20
6293 measured reflections	

### Refinement

**Table:**

Refinement on *F*^2^	Primary atom site location: structure-invariant direct methods
Least-squares matrix: full	Secondary atom site location: difference Fourier map
*R*[*F*^2^ > 2σ(*F*^2^)] = 0.040	Hydrogen site location: inferred from neighbouring sites
*wR*(*F*^2^) = 0.119	H atoms treated by a mixture of independent and constrained refinement
*S* = 1.05	*w* = 1/[σ^2^(*F*_o_^2^) + (0.0796*P*)^2^ + 0.2871*P*] where *P* = (*F*_o_^2^ + 2*F*_c_^2^)/3
1794 reflections	(Δ/σ)_max_ = 0.001
240 parameters	Δρ_max_ = 0.19 e Å^−^^3^
24 restraints	Δρ_min_ = −0.23 e Å^−^^3^

### Fractional atomic coordinates and isotropic or equivalent isotropic displacement parameters (Å^2^)

**Table:**

	*x*	*y*	*z*	*U*_iso_*/*U*_eq_	Occ. (<1)
N1	0.9898 (3)	0.26050 (14)	1.05516 (10)	0.0294 (4)	0.625
C1'	0.9898 (3)	0.26050 (14)	1.05516 (10)	0.0294 (4)	0.375
N2	1.0082 (3)	0.28041 (13)	1.18789 (10)	0.0339 (4)	
H1	1.035 (5)	0.3483 (10)	1.1827 (17)	0.052 (8)*	
H2	1.031 (4)	0.254 (2)	1.2335 (9)	0.045 (8)*	
N3	0.9475 (4)	0.02407 (14)	1.26992 (10)	0.0396 (5)	
C1	0.9186 (4)	−0.07603 (16)	1.08021 (11)	0.0369 (5)	
C2	1.0684 (4)	−0.13801 (17)	1.10772 (12)	0.0414 (6)	
H2A	1.1883	−0.1063	1.1200	0.050*	
C3	1.0422 (5)	−0.24706 (17)	1.11721 (12)	0.0463 (7)	
H3	1.1450	−0.2896	1.1355	0.056*	
C4	0.8687 (5)	−0.29307 (17)	1.10017 (12)	0.0500 (8)	
H4	0.8513	−0.3671	1.1075	0.060*	
C5	0.7187 (5)	−0.23191 (18)	1.07229 (14)	0.0521 (7)	
H5	0.5990	−0.2641	1.0603	0.063*	
C6	0.7439 (5)	−0.12301 (18)	1.06183 (13)	0.0478 (6)	
H6	0.6418	−0.0811	1.0422	0.057*	
C7	0.9453 (4)	0.04111 (15)	1.06991 (11)	0.0336 (5)	
C8	0.9536 (4)	0.08663 (16)	0.99730 (11)	0.0391 (6)	
C9	0.9790 (10)	0.0170 (5)	0.9251 (4)	0.0390 (15)	0.50
H9A	1.0343	−0.0525	0.9396	0.047*	0.50
H9B	0.8524	0.0043	0.9010	0.047*	0.50
C10	1.1119 (9)	0.0727 (5)	0.8684 (3)	0.0440 (15)	0.50
H10A	1.1216	0.0310	0.8206	0.053*	0.50
H10B	1.2422	0.0801	0.8905	0.053*	0.50
C9'	0.9024 (9)	0.0270 (5)	0.9245 (4)	0.0390 (15)	0.50
H9'C	0.9220	−0.0500	0.9322	0.047*	0.50
H9'D	0.7656	0.0390	0.9117	0.047*	0.50
C10'	1.0290 (10)	0.0659 (5)	0.8592 (3)	0.0440 (15)	0.50
H10C	1.1628	0.0426	0.8683	0.053*	0.50
H10D	0.9847	0.0335	0.8108	0.053*	0.50
C11	1.0247 (5)	0.18419 (19)	0.85163 (13)	0.0485 (7)	
C12	1.0305 (5)	0.2290 (2)	0.77912 (15)	0.0561 (7)	
H12	1.0608	0.1859	0.7362	0.067*	
C13	0.9929 (5)	0.3351 (3)	0.76887 (17)	0.0606 (8)	
H13	0.9963	0.3651	0.7191	0.073*	
C14	0.9497 (5)	0.3981 (3)	0.8318 (2)	0.0699 (10)	
H14	0.9246	0.4716	0.8250	0.084*	
C15	0.9431 (5)	0.3542 (2)	0.90409 (18)	0.0593 (9)	
H15	0.9134	0.3976	0.9469	0.071*	
C16	0.9798 (4)	0.24669 (16)	0.91469 (12)	0.0355 (5)	
C17	0.9738 (3)	0.19760 (15)	0.99194 (12)	0.0331 (5)	
C18	0.9871 (3)	0.21628 (14)	1.12653 (11)	0.0281 (4)	
C19	0.9622 (3)	0.10550 (15)	1.13457 (11)	0.0291 (4)	
C20	0.9531 (4)	0.05995 (15)	1.20987 (11)	0.0308 (5)	
N4	1.0072 (11)	0.4629 (4)	1.0526 (3)	0.0471 (15)	0.375
C21	0.9965 (10)	0.3721 (4)	1.0492 (3)	0.0337 (12)	0.375

### Atomic displacement parameters (Å^2^)

**Table:**

	*U*^11^	*U*^22^	*U*^33^	*U*^12^	*U*^13^	*U*^23^
N1	0.0427 (10)	0.0207 (8)	0.0249 (8)	0.0010 (8)	0.0045 (8)	0.0015 (7)
C1'	0.0427 (10)	0.0207 (8)	0.0249 (8)	0.0010 (8)	0.0045 (8)	0.0015 (7)
N2	0.0607 (12)	0.0210 (7)	0.0200 (7)	−0.0002 (9)	−0.0011 (8)	−0.0010 (6)
N3	0.0672 (14)	0.0253 (8)	0.0263 (8)	0.0000 (9)	−0.0015 (9)	0.0044 (7)
C1	0.0703 (16)	0.0214 (9)	0.0189 (8)	−0.0041 (10)	−0.0016 (10)	0.0008 (7)
C2	0.0705 (16)	0.0260 (10)	0.0277 (9)	0.0011 (11)	−0.0012 (11)	−0.0002 (8)
C3	0.087 (2)	0.0257 (10)	0.0259 (10)	0.0058 (12)	0.0027 (12)	0.0009 (8)
C4	0.106 (2)	0.0201 (9)	0.0239 (10)	−0.0060 (13)	0.0035 (12)	0.0016 (8)
C5	0.089 (2)	0.0300 (10)	0.0370 (12)	−0.0174 (13)	−0.0096 (13)	0.0029 (10)
C6	0.0785 (18)	0.0283 (10)	0.0365 (11)	−0.0092 (12)	−0.0159 (13)	0.0053 (9)
C7	0.0539 (13)	0.0211 (9)	0.0258 (9)	−0.0025 (9)	−0.0007 (10)	0.0002 (8)
C8	0.0704 (16)	0.0228 (9)	0.0241 (9)	−0.0043 (11)	−0.0002 (11)	0.0017 (8)
C9	0.069 (4)	0.0245 (14)	0.0237 (10)	0.001 (3)	−0.002 (3)	0.0004 (10)
C10	0.082 (5)	0.0283 (13)	0.0211 (15)	−0.013 (3)	−0.001 (2)	−0.0071 (11)
C9'	0.069 (4)	0.0245 (14)	0.0237 (10)	0.001 (3)	−0.002 (3)	0.0004 (10)
C10'	0.082 (5)	0.0283 (13)	0.0211 (15)	−0.013 (3)	−0.001 (2)	−0.0071 (11)
C11	0.0804 (19)	0.0357 (11)	0.0293 (11)	−0.0080 (14)	0.0008 (12)	0.0083 (9)
C12	0.080 (2)	0.0562 (15)	0.0321 (12)	−0.0134 (16)	0.0003 (13)	0.0126 (11)
C13	0.0538 (14)	0.0720 (19)	0.0559 (16)	0.0019 (16)	0.0020 (14)	0.0404 (15)
C14	0.0675 (19)	0.0549 (16)	0.087 (2)	0.0299 (16)	0.0384 (18)	0.0452 (16)
C15	0.0674 (18)	0.0408 (13)	0.0698 (18)	0.0201 (14)	0.0372 (16)	0.0274 (13)
C16	0.0429 (11)	0.0291 (10)	0.0347 (11)	0.0004 (10)	0.0053 (9)	0.0096 (9)
C17	0.0456 (11)	0.0240 (9)	0.0297 (10)	0.0003 (10)	0.0041 (10)	0.0026 (8)
C18	0.0405 (11)	0.0215 (8)	0.0224 (9)	0.0005 (9)	0.0001 (8)	−0.0002 (7)
C19	0.0432 (11)	0.0224 (9)	0.0218 (9)	−0.0005 (9)	0.0001 (9)	0.0028 (7)
C20	0.0480 (12)	0.0195 (8)	0.0249 (9)	−0.0013 (9)	−0.0017 (9)	−0.0011 (7)
N4	0.094 (5)	0.021 (2)	0.026 (2)	−0.002 (3)	0.001 (3)	−0.0014 (18)
C21	0.055 (3)	0.027 (3)	0.018 (2)	0.003 (3)	0.005 (2)	−0.0001 (19)

### Geometric parameters (Å, °)

**Table:**

N1—C17	1.366 (3)	C9—H9B	0.9900
N1—C18	1.367 (2)	C10—C11	1.560 (7)
N2—C18	1.352 (2)	C10—H10A	0.9900
N2—H1	0.88 (1)	C10—H10B	0.9900
N2—H2	0.88 (1)	C9'—C10'	1.523 (7)
N3—C20	1.144 (3)	C9'—H9'C	0.9900
C1—C6	1.391 (4)	C9'—H9'D	0.9900
C1—C2	1.389 (4)	C10'—C11	1.498 (7)
C1—C7	1.499 (3)	C10'—H10C	0.9900
C2—C3	1.397 (3)	C10'—H10D	0.9900
C2—H2A	0.9500	C11—C12	1.389 (3)
C3—C4	1.373 (4)	C11—C16	1.391 (3)
C3—H3	0.9500	C12—C13	1.375 (4)
C4—C5	1.387 (4)	C12—H12	0.9500
C4—H4	0.9500	C13—C14	1.390 (5)
C5—C6	1.396 (3)	C13—H13	0.9500
C5—H5	0.9500	C14—C15	1.382 (4)
C6—H6	0.9500	C14—H14	0.9500
C7—C19	1.397 (3)	C15—C16	1.392 (3)
C7—C8	1.395 (3)	C15—H15	0.9500
C8—C17	1.410 (3)	C16—C17	1.487 (3)
C8—C9'	1.521 (7)	C18—C19	1.415 (2)
C8—C9	1.548 (7)	C19—C20	1.438 (3)
C9—C10	1.527 (7)	N4—C21	1.149 (7)
C9—H9A	0.9900		
			
C17—N1—C18	120.11 (16)	C10'—C9'—C8	109.5 (4)
C18—N2—H1	122 (2)	C10'—C9'—H9'C	109.8
C18—N2—H2	120.8 (19)	C8—C9'—H9'C	109.8
H1—N2—H2	115 (3)	C10'—C9'—H9'D	109.8
C6—C1—C2	119.8 (2)	C8—C9'—H9'D	109.8
C6—C1—C7	120.0 (2)	H9'C—C9'—H9'D	108.2
C2—C1—C7	120.2 (2)	C11—C10'—C9'	112.1 (5)
C1—C2—C3	119.8 (3)	C11—C10'—H10C	109.2
C1—C2—H2A	120.1	C9'—C10'—H10C	109.2
C3—C2—H2A	120.1	C11—C10'—H10D	109.2
C4—C3—C2	120.3 (3)	C9'—C10'—H10D	109.2
C4—C3—H3	119.8	H10C—C10'—H10D	107.9
C2—C3—H3	119.8	C12—C11—C16	120.0 (2)
C3—C4—C5	120.2 (2)	C12—C11—C10'	119.0 (3)
C3—C4—H4	119.9	C16—C11—C10'	119.9 (3)
C5—C4—H4	119.9	C12—C11—C10	121.8 (3)
C4—C5—C6	119.9 (3)	C16—C11—C10	116.7 (3)
C4—C5—H5	120.0	C13—C12—C11	120.6 (3)
C6—C5—H5	120.0	C13—C12—H12	119.7
C1—C6—C5	119.9 (3)	C11—C12—H12	119.7
C1—C6—H6	120.1	C12—C13—C14	119.6 (2)
C5—C6—H6	120.1	C12—C13—H13	120.2
C19—C7—C8	119.64 (17)	C14—C13—H13	120.2
C19—C7—C1	119.05 (17)	C15—C14—C13	120.1 (3)
C8—C7—C1	121.31 (17)	C15—C14—H14	119.9
C7—C8—C17	118.24 (18)	C13—C14—H14	119.9
C7—C8—C9'	123.3 (3)	C14—C15—C16	120.4 (3)
C17—C8—C9'	117.3 (3)	C14—C15—H15	119.8
C7—C8—C9	120.9 (3)	C16—C15—H15	119.8
C17—C8—C9	119.8 (3)	C15—C16—C11	119.2 (2)
C10—C9—C8	109.7 (5)	C15—C16—C17	121.4 (2)
C10—C9—H9A	109.7	C11—C16—C17	119.40 (18)
C8—C9—H9A	109.7	N1—C17—C8	122.06 (18)
C10—C9—H9B	109.7	N1—C17—C16	119.48 (18)
C8—C9—H9B	109.7	C8—C17—C16	118.46 (18)
H9A—C9—H9B	108.2	N2—C18—N1	118.65 (16)
C9—C10—C11	107.5 (5)	N2—C18—C19	121.67 (16)
C9—C10—H10A	110.2	N1—C18—C19	119.68 (17)
C11—C10—H10A	110.2	C7—C19—C18	120.24 (17)
C9—C10—H10B	110.2	C7—C19—C20	120.37 (17)
C11—C10—H10B	110.2	C18—C19—C20	119.39 (17)
H10A—C10—H10B	108.5	N3—C20—C19	179.3 (3)
			
C6—C1—C2—C3	−0.4 (3)	C10—C11—C12—C13	−165.4 (4)
C7—C1—C2—C3	179.9 (2)	C11—C12—C13—C14	0.4 (5)
C1—C2—C3—C4	−0.6 (3)	C12—C13—C14—C15	−0.5 (5)
C2—C3—C4—C5	1.0 (4)	C13—C14—C15—C16	0.1 (5)
C3—C4—C5—C6	−0.3 (4)	C14—C15—C16—C11	0.5 (5)
C2—C1—C6—C5	1.1 (4)	C14—C15—C16—C17	179.9 (3)
C7—C1—C6—C5	−179.3 (2)	C12—C11—C16—C15	−0.7 (5)
C4—C5—C6—C1	−0.7 (4)	C10'—C11—C16—C15	−168.7 (4)
C6—C1—C7—C19	110.6 (3)	C10—C11—C16—C15	165.6 (3)
C2—C1—C7—C19	−69.8 (3)	C12—C11—C16—C17	179.9 (3)
C6—C1—C7—C8	−69.5 (3)	C10'—C11—C16—C17	11.8 (5)
C2—C1—C7—C8	110.1 (3)	C10—C11—C16—C17	−13.8 (4)
C19—C7—C8—C17	−1.7 (4)	C18—N1—C17—C8	0.2 (4)
C1—C7—C8—C17	178.4 (2)	C18—N1—C17—C16	−179.2 (2)
C19—C7—C8—C9'	−169.0 (4)	C7—C8—C17—N1	1.5 (4)
C1—C7—C8—C9'	11.0 (5)	C9'—C8—C17—N1	169.6 (3)
C19—C7—C8—C9	166.8 (4)	C9—C8—C17—N1	−167.1 (4)
C1—C7—C8—C9	−13.1 (5)	C7—C8—C17—C16	−179.1 (2)
C7—C8—C9—C10	−141.5 (4)	C9'—C8—C17—C16	−11.0 (4)
C17—C8—C9—C10	26.9 (6)	C9—C8—C17—C16	12.3 (5)
C9'—C8—C9—C10	115.4 (13)	C15—C16—C17—N1	−19.7 (4)
C8—C9—C10—C11	−55.9 (6)	C11—C16—C17—N1	159.7 (2)
C7—C8—C9'—C10'	−146.4 (4)	C15—C16—C17—C8	160.9 (3)
C17—C8—C9'—C10'	46.2 (6)	C11—C16—C17—C8	−19.7 (4)
C9—C8—C9'—C10'	−56.5 (11)	C17—N1—C18—N2	178.3 (2)
C8—C9'—C10'—C11	−52.0 (6)	C17—N1—C18—C19	−1.8 (3)
C9'—C10'—C11—C12	−143.3 (4)	C8—C7—C19—C18	0.2 (4)
C9'—C10'—C11—C16	24.9 (6)	C1—C7—C19—C18	−179.9 (2)
C9'—C10'—C11—C10	113.0 (11)	C8—C7—C19—C20	179.7 (2)
C9—C10—C11—C12	−142.0 (4)	C1—C7—C19—C20	−0.4 (3)
C9—C10—C11—C16	51.9 (5)	N2—C18—C19—C7	−178.5 (2)
C9—C10—C11—C10'	−52.2 (9)	N1—C18—C19—C7	1.6 (3)
C16—C11—C12—C13	0.2 (5)	N2—C18—C19—C20	2.0 (4)
C10'—C11—C12—C13	168.4 (4)	N1—C18—C19—C20	−177.9 (2)

### Hydrogen-bond geometry (Å, °)

**Table:**

*D*—H···*A*	*D*—H	H···*A*	*D*···*A*	*D*—H···*A*
N2—H1···N3^i^	0.88 (1)	2.37 (2)	3.175 (2)	152 (3)

Symmetry codes: (i) −*x*+2, *y*+1/2, −*z*+5/2.
